# When Benign Mimics Malignancy: Parapharyngeal Oncocytoma in a Patient With Marginal Zone Lymphoma

**DOI:** 10.1002/ccr3.72045

**Published:** 2026-02-11

**Authors:** Somaya Al Kiswani, Tala Al‐Hyasat, Hossam Salameh, Ziyad Alqasem, Mohammed AbuBaha, Omar Sawafta, Abdullah Nofal, Mohammad Z. Al‐Qaisi

**Affiliations:** ^1^ Radiology Department King Hussein Cancer Center Amman Jordan; ^2^ Department of Medicine An‐Najah National University Nablus Palestine; ^3^ Specialist Radiologist, Radiology Department Hamad Medical Corporation Doha Qatar

**Keywords:** atypical, FDG‐PET imaging, marginal zone lymphoma, Oncocytoma, parapharyngeal space tumor

## Abstract

Parapharyngeal oncocytomas are rare benign salivary gland tumors that can be challenging to distinguish from malignant lesions on imaging, particularly in patients with a history of lymphoma. This case underscores the importance of histopathological confirmation and multidisciplinary evaluation in managing deep‐neck masses, particularly when imaging may falsely suggest malignancy.

## Graphical abstract

Chronological overview of clinical presentation, diagnostic procedures, and longitudinal PET imaging demonstrating evolving metabolic activity of a parapharyngeal oncocytoma depicting the patient's clinical journey diagnostic evaluation, pathological confirmation of parapharyngeal oncocytoma, and subsequent follow‐up demonstrating disease stability.

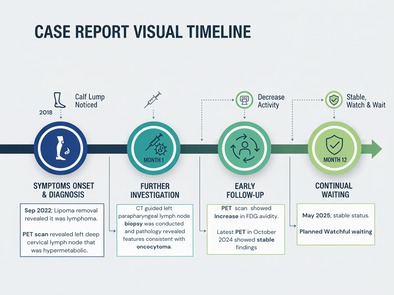

## 1 Introduction

In the current World Health Organization framework, oncocytotic lesions are classified as nodular oncocytic hyperplasia, oncocytoma, and oncocytic carcinoma [1]. Oncocytomas are rare benign epithelial neoplasms that are almost exclusively composed of oncocytes, which are large polygonal cells with intensely eosinophilic, granular cytoplasm due to marked mitochondrial proliferation. About 78%–84% of instances of salivary‐gland oncocytomas occur in the parotid gland, and they make up 0.5%–1.5% of all salivary tumors [2]. Despite being histologically benign, oncocytomas may be difficult to diagnose due to their similar imaging appearances to other parapharyngeal and parotid entities [3, 4].

An additional level of complication is introduced by functional imaging. Despite benign histology, oncocytic tumors may exhibit disproportionately high metabolic activity on FDG‐PET, leading to false‐positive perceptions of malignancy [5, 6]. In order to prevent overtreatment and to arrange adequate, often multidisciplinary care, correlation with anatomic imaging and, critically, tissue diagnosis is essential.

In this context, we present a rare parapharyngeal oncocytoma identified during monitoring in a patient with a history of indolent lymphoma. The case highlights essential insights for doctors overseeing deep‐neck masses: maintain a broad differential for PPS lesions, including benign salivary oncocytoma; be aware that FDG uptake can be falsely positive in oncocytic tumors; and prioritize histopathological confirmation and multidisciplinary discussion when imaging and clinical context raise concerns for malignancy.

## 2 Case History and Examination

A 60‐year‐old man with a background of hypertension and prediabetes first noted a slowly enlarging left calf subcutaneous mass in 2018, which gradually increased in size over several years. He did not seek medical attention until September 2022, when the lesion was excised at an outside institution under the presumptive diagnosis of lipoma. Histopathologic evaluation revealed low‐grade B‐cell lymphoma, later reviewed at a tertiary center and classified as marginal zone lymphoma.

**FIGURE 1:**
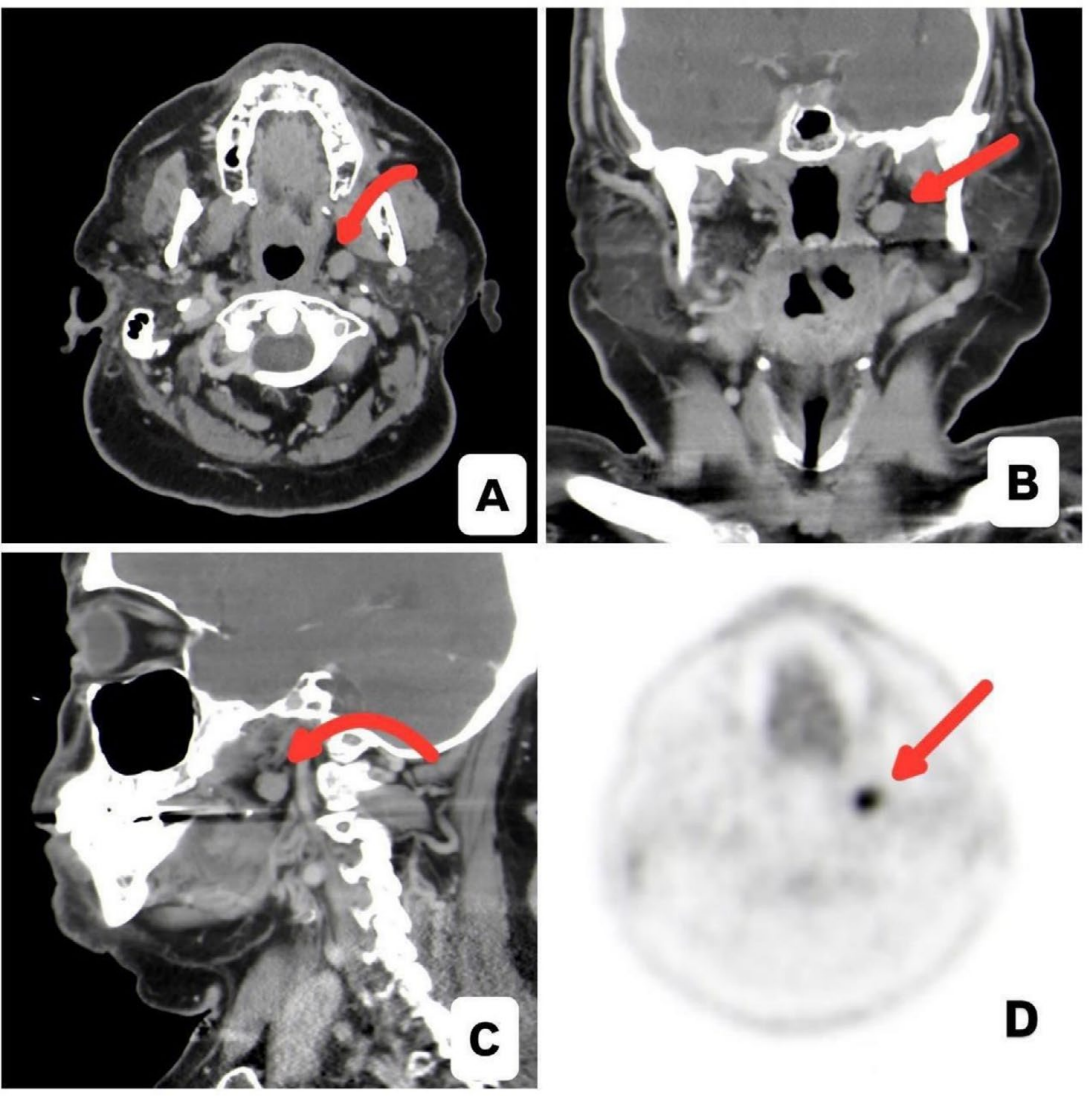
(A–C) Contrast‐enhanced CT images of the neck (A: Axial, B: Coronal, C: Sagittal) demonstrate a small, well‐defined left parapharyngeal nodular lesion, initially suspected to represent an abnormal lymph node. The lesion measures approximately 0.9 cm and shows soft‐tissue attenuation without aggressive features, surrounding fat stranding, or mass effect on adjacent structures. (D) Fused PET/CT image reveals marked FDG avidity within the previously suspected lymph node (SUVmax 9.7), significantly above physiologic background activity in the region.

A staging FDG‐PET/CT performed in October 2022 demonstrated a hypermetabolic left deep cervical/parapharyngeal lymph node with an SUVmax of 9.7, with no evidence of other systemic disease (Figure 1).

The patient remained entirely asymptomatic, reporting no B‐symptoms, airway compromise, dysphagia, or head‐and‐neck complaints. Nasopharyngolaryngoscopy demonstrated normal mobile vocal cords and no mucosal abnormalities, and routine laboratory studies, including CBC, LDH, ESR, and β2‐microglobulin, were within normal limits.

A contrast‐enhanced CT scan of the neck in November 2022 demonstrated a 1 cm well‐circumscribed parapharyngeal lesion deep to the parotid gland. Given the lesion's deep location and proximity to major vascular structures, percutaneous biopsy was initially deferred. However, follow‐up FDG‐PET/CT performed in February 2023 demonstrated interval increase in metabolic activity, with the parapharyngeal lesion reaching an SUVmax of 17.7, raising concern for possible lymphoma involvement or transformation despite stable lesion size.

Subsequently, a CT‐guided core needle biopsy was successfully performed on 7 March 2023 using an 18‐gauge needle. Histopathologic examination demonstrated oncocytic epithelial cells consistent with oncocytoma, with no evidence of lymphoma.

The case was reviewed in a multidisciplinary setting involving hematology, radiology, otolaryngology, and head and neck surgery. In view of the benign pathology, lack of symptoms, and high surgical morbidity associated with the parapharyngeal location, active surveillance was recommended. Serial clinical assessments and imaging studies through October 2024 demonstrated stable lesion size and metabolic activity.

As of May 2025, the patient remains clinically stable and asymptomatic, with no evidence of disease progression and continues under routine multidisciplinary surveillance.

## 3 Differential Diagnosis

The combination of rising metabolic activity and the patient's lymphoma background raised concern for recurrent or transformed MZL; however, the lesion's well‐defined appearance was atypical for lymphoma relapse.

Radiologic and clinical features supported a broad differential diagnosis that included salivary‐type parapharyngeal tumors such as oncocytoma, pleomorphic adenoma, or Warthin tumor, as well as neurogenic neoplasms like schwannoma or paraganglioma.

Metastatic lymphadenopathy was also considered, along with the possibility of a second primary neoplasm, although benign epithelial tumors such as oncocytoma are rarely encountered in this context. Overall, the differential demonstrated significant overlap, emphasizing the need for tissue confirmation.

**FIGURE 2:**
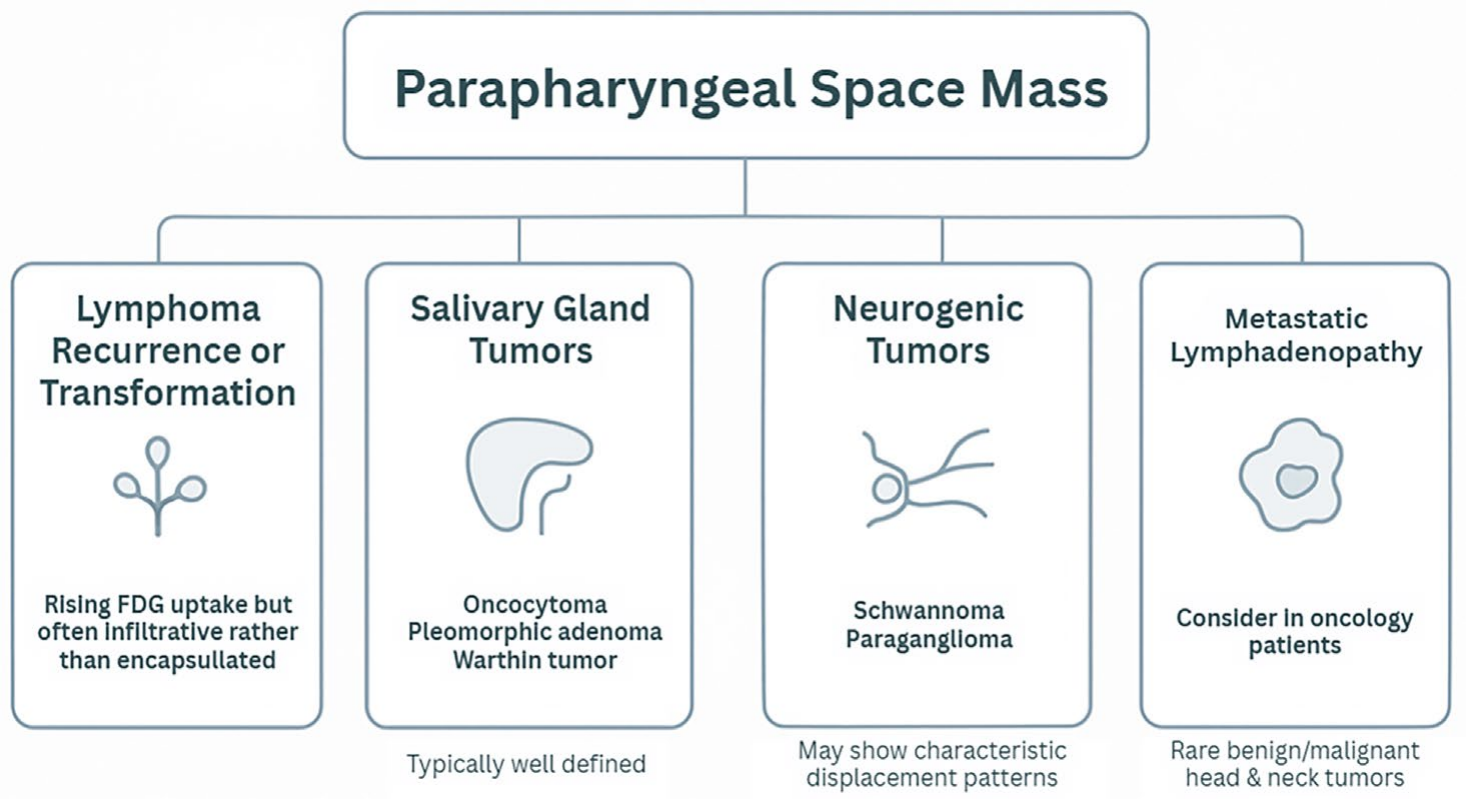
Schematic differential diagnosis overview for a parapharyngeal space mass, illustrating key categories, including lymphoma recurrence or transformation, salivary gland tumors, neurogenic tumors, metastatic lymphadenopathy, and second primary neoplasms.

Summarized in Figure 2 are differential diagnoses for this patient.

## 4 Investigations

Initial imaging with CT of the neck revealed a well‐defined parapharyngeal lesion without infiltrative features. Serial PET/CT scans performed between 2022 and 2024 demonstrated persistently high FDG uptake with a rising SUVmax, although the lesion's size and morphology remained stable.

Endoscopic examination of the upper aerodigestive tract was unremarkable, and laboratory studies remained normal throughout follow‐up. Although biopsy was initially deferred due to the lesion's proximity to major vascular structures, increasing metabolic activity prompted CT‐guided core needle biopsy in March 2023. Histopathologic examination demonstrated oncocytic epithelial cells with abundant granular cytoplasm and low proliferative activity, consistent with oncocytoma. Immunohistochemistry was positive for P63 and negative for PAX‐8, PAX‐5, chromogranin, S‐100, HPV, and calponin, confirming the benign salivary‐type neoplasm.

## 5 Treatment

The findings were reviewed in a multidisciplinary head and neck tumor board involving ENT, hematology, radiology, and pathology specialists. Considering the benign pathology, the lesion's stability, and the patient's asymptomatic course, surgery was not recommended due to the parapharyngeal space's high operative morbidity. The consensus was to pursue active surveillance with continued radiologic and clinical monitoring. No additional therapeutic intervention was deemed necessary.

## 6 Conclusion and Results (Outcome and Follow‐Up)

This case illustrates the diagnostic challenge posed by parapharyngeal oncocytoma, which can exhibit markedly elevated FDG uptake and closely mimic malignant relapse in patients with a history of lymphoma. Histopathologic confirmation was essential to avoid misdiagnosis and prevent unnecessary or potentially morbid treatment. Serial PET/CT imaging from May 2023 through October 2024 demonstrated stable metabolic activity in the parapharyngeal lesion, and additional subcutaneous nodules in the thighs and pelvis also remained stable and compatible with the patient's known indolent MZL. As of May 2025, the patient remains clinically stable, asymptomatic, and without evidence of progression, continuing under routine surveillance by the hematology and ENT services.

**FIGURE 3:**
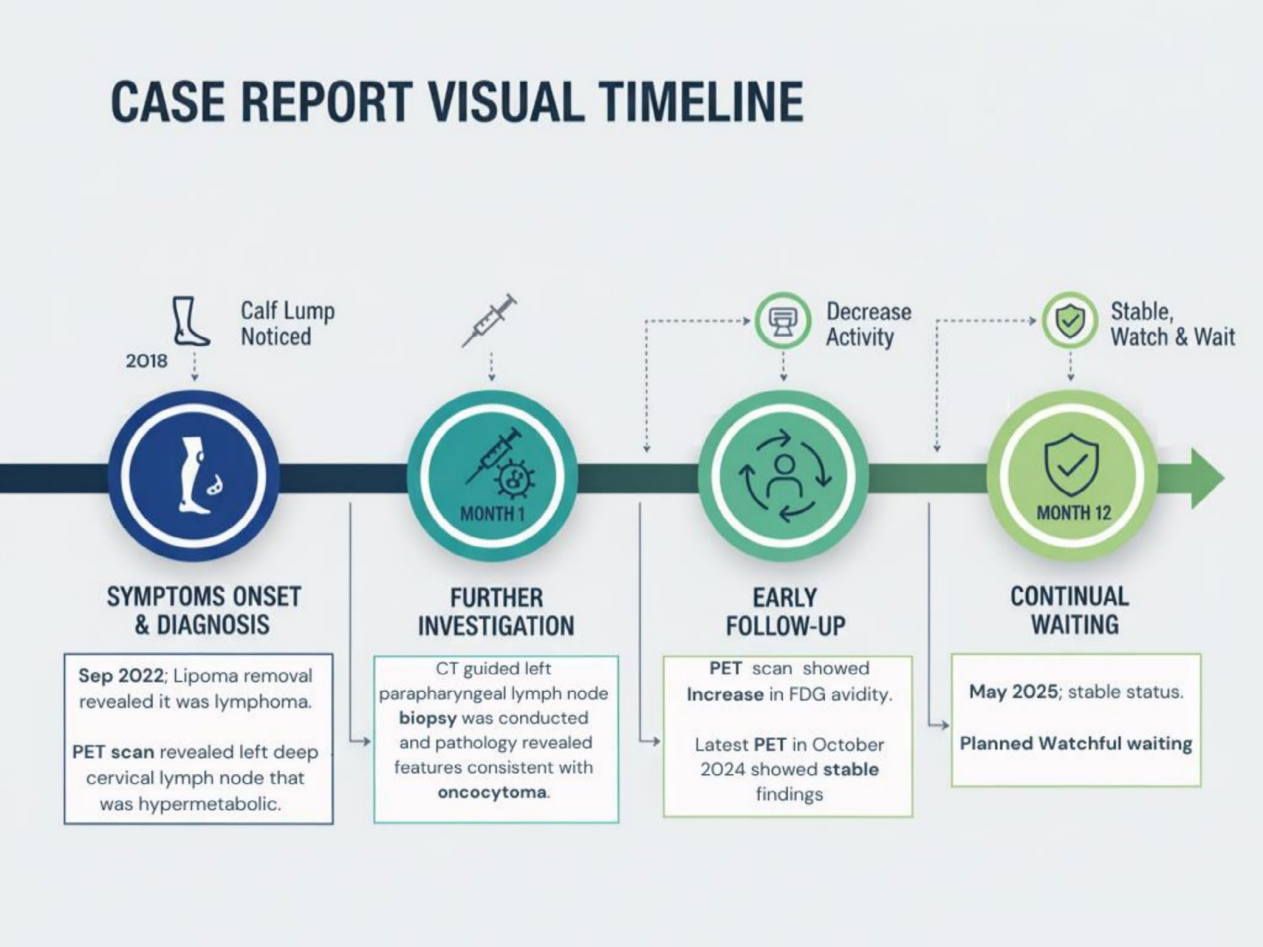
Visual timeline of the clinical course.

A visual timeline of the patient's clinical course is illustrated in Figure 3.

## 7 Discussion

Parapharyngeal space (PPS) tumors are rare, accounting for 0.5%–1% of head and neck neoplasms, and arise from vascular, neurogenic, or salivary structures in the deep corridor from the skull base to the hyoid [7, 8, 9]. Their diagnosis and surgical management are challenging due to proximity to critical neurovascular structures and frequent deep‐lobe parotid or minor salivary gland origins [7, 8, 9]. PPS oncocytomas are particularly uncommon, usually representing deep‐lobe parotid tumors extending into the PPS or, less often, true non‐parotid oncocytomas from minor salivary tissue [10].

Oncocytoma is a benign neoplasm of the head and neck, including nodular oncocytic hyperplasia and oncocytic carcinoma [11]. It consists of epithelial cells with eosinophilic granular cytoplasm (oncocytes), forming solid sheets, nests, islands, or duct‐like structures [12, 13]. Clear‐cell variants contain cytoplasmic glycogen, typically confirmed by periodic acid–Schiff (PAS) staining [14]. In this case, PAS staining was not required as diagnosis was established based on characteristic histology and supportive immunohistochemistry.

In patients with prior lymphoma, a new head and neck mass raises concern for relapse. Recurrent marginal zone lymphoma (MZL) usually presents with nodal or extranodal lesions, constitutional “B” symptoms, and infiltrative imaging findings with moderate‐to‐intense FDG uptake [15, 16]. In our patient, the lesion was well‐circumscribed and encapsulated, without systemic symptoms, which is atypical for lymphoma recurrence. Secondary neoplasms can occur after lymphoma therapy, including thyroid carcinoma, sarcomas, and salivary gland tumors [17, 18]; however, benign epithelial tumors like oncocytoma are extremely rare, and any association with prior lymphoma or cytogenetic anomalies remains speculative.

Beyond relapse and secondary malignancy, the parapharyngeal space (PPS) hosts a heterogeneous spectrum of benign and malignant lesions. Approximately 80% of PPS tumors are benign, most frequently of salivary, neurogenic, or vascular origin [7]. Salivary tumors, such as pleomorphic adenoma, Warthin tumor, or oncocytoma, typically arise from the deep lobe of the parotid gland and are usually located in the prestyloid space, presenting as slowly enlarging, non‐tender masses. On imaging, these tumors generally displace the internal carotid artery posteriorly. Neurogenic tumors, including schwannomas, often arise in the post‐styloid space and characteristically displace the carotid sheath anteriorly. Paragangliomas also most commonly occupy the post‐styloid space and displace the carotid sheath anteriorly; however, the classic “lyre sign” is specific to carotid body tumors rather than paragangliomas as a broader group [19]. Vascular lesions demonstrate avid contrast enhancement and flow voids on MRI, unlike the solid enhancement pattern of oncocytomas [20]. Table 1 summarizes the findings of different tumors in the PPS.

**TABLE 1:** Major types of parapharyngeal space (PPS) tumors.

Tumor Type	Common examples	Origin/Site of arising	Typical clinical presentation	Characteristic imaging findings	Key distinguishing features
Salivary Tumors	Pleomorphic adenoma, Warthin tumor, Oncocytoma	Deep lobe of the parotid gland extending into the PPS	Slowly enlarging, non‐tender, well‐circumscribed mass; usually asymptomatic	Well‐defined, encapsulated, solid lesion with homogeneous enhancement on CT/MRI	Benign epithelial origin; often difficult to distinguish radiologically without biopsy
Neurogenic Tumors	Schwannoma, Paraganglioma	Derived from neural sheath (schwannoma) or paraganglia (paraganglioma)	Painless neck mass; may cause cranial nerve deficits if large	Smooth margins; schwannomas displace carotid sheath posteriorly; paragangliomas separate internal and external carotid arteries (the “lyre sign”)	Location and vessel displacement patterns help differentiate subtypes
Vascular Tumors/Lesions	Hemangioma, Vascular malformation	Arising from vascular or venolymphatic structures	Pulsatile or compressible mass; may enlarge with Valsalva maneuver	Marked, avid contrast enhancement; flow voids on MRI due to high vascularity	Strong enhancement and flow voids differentiate from solid lesions like oncocytoma

Imaging characteristics, therefore, provide initial diagnostic guidance: lymphoma typically presents as diffuse or infiltrative tissue with homogeneous enhancement and nodal chains, whereas oncocytoma appears as a well‐defined, encapsulated, avidly enhancing lesion due to its mitochondrial density [4]. Nevertheless, definitive diagnosis requires histopathological confirmation, as overlapping radiologic features can lead to misclassification. Table 2 demonstrates different tumor characteristics.

**TABLE 2:** Differential features distinguishing lymphoma recurrence from oncocytoma and other parapharyngeal space tumors.

Feature	Lymphoma recurrence (Marginal zone/B‐cell type)	Oncocytoma (Salivary‐type benign tumor)	Other parapharyngeal tumors (Neurogenic/Vascular)
Nature	Malignant hematolymphoid neoplasm (B‐cell origin)	Benign epithelial neoplasm of salivary origin	Usually benign; neurogenic (schwannoma, paraganglioma) or vascular (hemangioma)
Typical Patient Profile	Prior history of lymphoma; middle‐aged to elderly; may have prior chemo/radiotherapy	Older adults; extremely rare in children; no systemic symptoms	Wide age range depending on subtype; paragangliomas are more common in adults
Clinical Presentation	New or enlarging nodal or extranodal mass; may present with B‐symptoms (fever, night sweats, weight loss)	Slow‐growing, non‐tender, well‐circumscribed mass; usually asymptomatic	Painless mass; paragangliomas may cause a pulsatile mass or cranial nerve symptoms
Imaging Features (CT/MRI/PET)	Ill‐defined, infiltrative, homogeneous enhancement; moderate–intense FDG uptake on PET	Well‐defined, encapsulated, solid lesion; avid but focal FDG uptake due to mitochondrial content	‐ Schwannoma: smooth, T2‐hyperintense, displaces carotid sheath posteriorly—Paraganglioma: separates internal and external carotids (“lyre sign”)—Vascular: marked enhancement, flow voids on MRI
Systemic Involvement	Often part of disseminated disease; nodal chains or extranodal spread	Localized to the zparapharyngeal/deep parotid region	Usually localized, rarely systemic

## Author Contributions

**Somaya Al Kiswani:** writing – original draft. **Tala Al‐Hyasat:** writing – original draft. **Hossam Salameh:** project administration, visualization, writing – original draft. **Ziyad Alqasem:** writing – original draft. **Mohammed AbuBaha:** writing – original draft. **Omar Sawafta:** writing – original draft. **Abdullah Nofal:** investigation. **Mohammad Z. Al‐Qaisi:** supervision.

## Funding

The authors have nothing to report.

## Consent

Written informed consent was obtained from a legally authorized representative(s) for anonymized patient information to be published in this article.

## Conflicts of Interest

The authors declare no conflicts of interest.

## Data Availability

The data used to support the findings of this study are available from the corresponding author upon reasonable request.
